# Economics of Philanthropy

**Published:** 1898-03-19

**Authors:** 


### The Institutional Workshop.

### ECONOMICS OF PHILANTHROPY.

### V.—FRIENDLY SOCIETIES.

While it is conceivable that under a system of small holdings, especially if these belonged to the cultivators, land which in England is now falling out of cultivation might be made profitable, and that which is now cultivated become more productive, it is very generally alleged that English husbandry lacks not only energy, but guidance. The same is urged of all branches of industry in which our country had once a pre-eminence, if not a monopoly, and in which it no longer keeps so far ahead of other nations. To the perception of the fact that Englishmen are not so well educated in the principles and methods of their trade, we owe the present development of technical education. It is difficult to say how much money is spent altogether on this, for to disentangle the various sources of income, local and national, of the various schools would take too much space. But a recent student* of the question estimates that the total expenditure on technical education cannot be less than two millions sterling. These schools embrace every variety of subject. Some are content only to instruct in the chief industries of the district in which they are situated, while others take a larger view of their duties, and give instruction in the sciences which underly the useful arts. While both are necessary, it is not difficult to perceive that the former will send their pupils out, skilled, indeed, in such operations and processes as are now in vogue, rather than fitted to develop new methods of work. It is thus obvious that while mere manual training is by no means to be despised, it does not fill the entire field of technical instruction. An improvement in machinery, an economy in production, may mean all the difference between profit and loss. It is by superiority in scientific knowledge, as much as by working longer hours and being content with lower wages, that the much-dreaded German gains the advantage. "While the chemical industry of Tyneside languishes," says a recent writer, "and the older sources of industry, which are rapidly becoming dried up, have not as yet received a substitute in new directions, towns have sprung up in Germany which owe their prosperity to the utilisation of chemical bye-products for commercial purposes." American competition, which no one can suppose is made possible by the low wages or small demands of the worker, may be traced to the remarkable ingenuity of Americans in inventing machinery, and the almost equally remarkable capacity which the working men of the West show in the management of the most complicated machines, even when these are not familiar to them. We have spoken of these two competitors because they are the best-known bugbears of the British workman; but, as a matter of fact, it is possible that in the near future we may have more cause to fear the competition of Russia. The writer we have already quoted says: "In the years 1880—1892 all branches of industry, except that of leather and similar animal products, showed an enormous increase in value. Textile fabrics, for instance, increased 38 per cent., metals 76 per cent., and chemical products 130 per cent. The progress made by Russia in engineering manufacture is such as to cause our own manufacturers serious misgivings."

* Mr. W. Chater, in the *Journal of Finance* for November, 1897, to whom the writer is indebted for the other quotations on the subject given in this article.

In face of such statements as these, we must regard money spent on technical education as devoted to a most philanthropic object. This does not of itself prove that it has been well spent; but we fully believe that in the result it will be shown to have been so. Only it must be remembered that it is not enough for the technical school to give the training that was formerly given in the workshop—and, indeed, it may be doubted if any school can fully compensate for that —but must so instruct the pupil in the science underlying his work that he will be able to take advantage of new discoveries as they arise.

Lord Armstrong, in an article entitled "The Vague Cry for Technical Education," which appeared in the *Nineteenth Century* for 1888, declared that no outside education could make up for the lack of workshop experience. And this is obvious. It is during his apprenticeship, when he is looking on at work as much as working himself, that a lad learns the handling of tools, and acquires deftness in their use. Manual training must always be given, in the ultimate resort, in the workshop; but technical training includes more than that, and failure results from confining it solely to this department. Those who conduct evening technical classes, to which young men come on their own initiative, after going to work and finding out what their needs are, and what subjects will best help them up the ladder of life, these teachers will tell you that their pupils come for instruction in higher arithmetic, and mathematics; in chemistry, in machine-drawing, in physics, and the like; in short, in the sciences which underlie their daily work, and on which every improvement in working is founded. But pure scientific knowledge is not enough, though it is essential. The leading men in a commercial nation will always be the men who can turn science to practical account in the advance of industry. The development of such men is the triumph of technical education; and with free elementary education, technical education largely subsidised, and bursaries and scholarships which lead from the one to the other, there is no reason why the poorest in the land, if he be possessed of ability enough, should not attain a position of comfort and independence by which both he and his country will profit. Those who lack either the brain or the will to take advantage of their opportunities must remain, in the future as in the past, the hewers of wood and the drawers of water. It is not in mortal power to change the differences between one man's capacity and another; but it is possible, and it should be the aim of well-directed philanthropy, to give the clever man the chance of using his ability to the best advantage; and the one who is left behind in the race may comfort himself by the thought that there has never been a useful discovery made in art, science, or industry, which has not benefited the mass of the people, as well as enriched those who introduced it.

It may be objected that what we have been speaking of is business, not philanthropy. To reply that a well-managed business is a successful experiment in philanthropy would imply approval of the rule of every Gradgrind in the kingdom, which certainly is not our desire; but as the aim of philanthropy is to raise the condition of the poor, we must give the highest place to those schemes which most fully tend to improve permanently the position of those whom they reach. But we turn now to a means of rendering the poor more independent, which most people will regard as more directly philanthropical, while, as a matter of fact, it owes less to the benefactions of the rich than does technical education. We allude to those benefit and friendly societies, which are formed by workmen themselves, either within the limits of their own trade, or on a larger scale. The joining such a society shows in the man who does it, that self-denial, that perception of the importance of the future, which is one of the first essentials of progress, which is, indeed, the thing that first differentiates savage from civilised peoples. That these societies are more popular with the working class than even the Post Office Savings' Bank is quite comprehensible. While money invested with them is sunk absolutely, they offer a larger income during such emergencies as workers look forward to, than could be obtained from a much larger capital, if it were merely deposited in a savings bank. A provision against sickness and old age of reasonable amount is, to working people, of more importance than the possession of a small capital, even though the latter be their own to bequeath to their children. The shilling or sixpence a week which they can contribute to a benefit society without seriously inconveniencing themselves brings a better return than the same sum would afford if invested in any other way. These societies own a large capital, and are, as a rule, well managed. The total membership is estimated at 10,000,000, and their funds amount to £90,000,000 sterling.

In the benefit societies associated with particular trades a stronger feeling of brotherhood comes in than in the larger societies. Each member perceives that the good of one is the good of all. This feeling, though most honourable in itself, has perhaps had in some ways an unfortunate effect. This is not the place in which to speak of strikes, their righteousness or unrighteousness, their wisdom or unwisdom. But if the resources of trades unions are to be expended in them the part of the income which is to be devoted to such struggles should be kept entirely separate from that which is intended for the benefit of aged or infirm members. The funds of the insurance society should not be deposited in the war-chest. It is cruel for a man who has for years entrusted his savings to the care of his trade union, in the expectation that they would be forthcoming for his use in sickness or old age, to find that they have been dissipated in a fight with the employers which, perhaps, he personally disapproved of. To act long on this principle may in the end destroy the power of the unions by keeping out of them the more prudent of the workers. And this would be a pity, for the benefit funds of a trade union are usually well expended. Being under the management of the local leaders, who know personally the character, as well as the needs of those who ask for sick or old age pay, they are less likely to be misapplied than funds the spending of which cannot be so carefully supervised. Malingering will receive small mercy at the hands of fellow-workers, while these same fellow-workers will show the greatest kindness in cases, however prolonged, of real ill-health.

Many friendly societies take only male members; a few include both men and women. So far as we know, there is only one such society for women only. That is one well known to readers of The Hospital—the Royal National Pension Fund for Nurses. It is needless to recapitulate here its provisions; but it shows how much can be done by, and will be done for, women who are by no means exceedingly well paid if they are encouraged to save, and are sure that their savings will be managed by those who have their welfare at heart, and are at the same time experienced men of business.

